# S-open Technique: A Novel, Sutureless, Open Laparoscopic Entry Technique

**DOI:** 10.7759/cureus.92836

**Published:** 2025-09-21

**Authors:** Pr Jihad El Anzaoui, Ali Akjay, Abdellah Baba Habib, Mohamed Menfaa, Aziz Zentar

**Affiliations:** 1 Urology, Faculty of Medicine, Université Sidi Mohamed Ben Abdellah, Fez, MAR; 2 Urology, Military Hospital Moulay Ismail, Meknes, MAR; 3 Gynecology, Military Hospital Moulay Ismail, Meknes, MAR; 4 General and Colorectal Surgery, Military Hospital Moulay Ismail, Meknes, MAR

**Keywords:** hasson technique, intra-abdominal surgery, laparoscopy, morocco, open entry technique

## Abstract

Safe abdominal entry remains a critical step in laparoscopic surgery, particularly in patients with a history of prior abdominal procedures. Traditional techniques, such as the Hasson open method, have been widely used, but they may require fascial suturing and carry a risk of entry-related complications.

This article evaluates the safety, feasibility, and outcomes of a new sutureless open laparoscopic entry technique developed and applied in our hospital.

A total of 76 patients underwent laparoscopic surgery using the proposed sutureless entry technique. Patients were classified based on their body mass index and the number of previous abdominal surgeries. Entry feasibility, complication rates, and postoperative outcomes were assessed.

Entry was successful in all patients. Complications were rare and primarily classified as Clavien-Dindo grade I. No cases of trocar-site hernia were recorded during a follow-up period ranging from four months to two years. The technique was applied effectively across three surgical specialties: urology, gynecology, and visceral surgery.

This novel sutureless open laparoscopic entry technique is a safe, simple, and reproducible alternative to traditional open methods. It can be especially useful in patients with previous abdominal surgery and may contribute to reducing operative time and postoperative complications.

## Introduction

Introducing the laparoscope is an essential and fundamental initial step in laparoscopic surgery. Without this step, the entire procedure cannot proceed. Although some authors have attempted to perform laparoscopic surgery without establishing pneumoperitoneum [1], visual control remains indispensable.

A wide variety of entry laparoscopic procedures exist in our practice [2], including the Veress needle approach, direct trocar insertion, and the open (Hasson) technique, each selected according to patient characteristics, prior surgical history, and the specific operative context.

The Hasson technique [3], also known as the open entry technique, is considered the most fundamental and widely utilized method for laparoscopic access. This approach is favored for its reproducibility, safety profile, and reduced risk of visceral or vascular injury compared to blind entry techniques, but remains challenging in cases of morbid obesity or scarred abdomen.

These two situations require a reliable, rapid, and safe approach. Here, we describe a simple, fast, reliable, and reproducible technique applicable to any type of abdomen.

## Technical report

**Figure 1:**
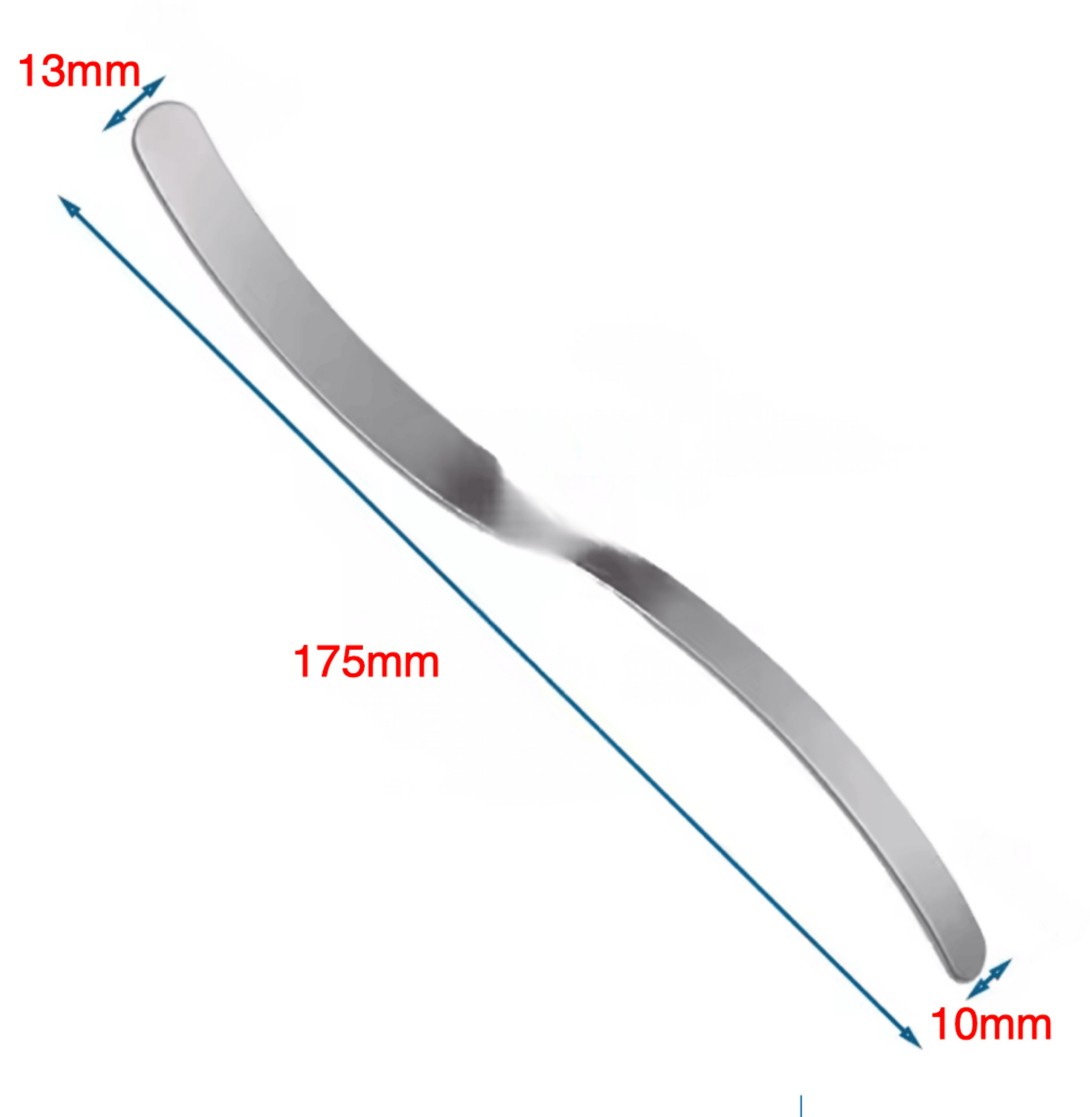
A special S-shaped retractor with two different extremities. The 10 mm extremity is used to enter the peritoneal cavity via a small hole in the aponeurosis and peritoneum that can be left open at the end of the procedure.

**Figure 2:**
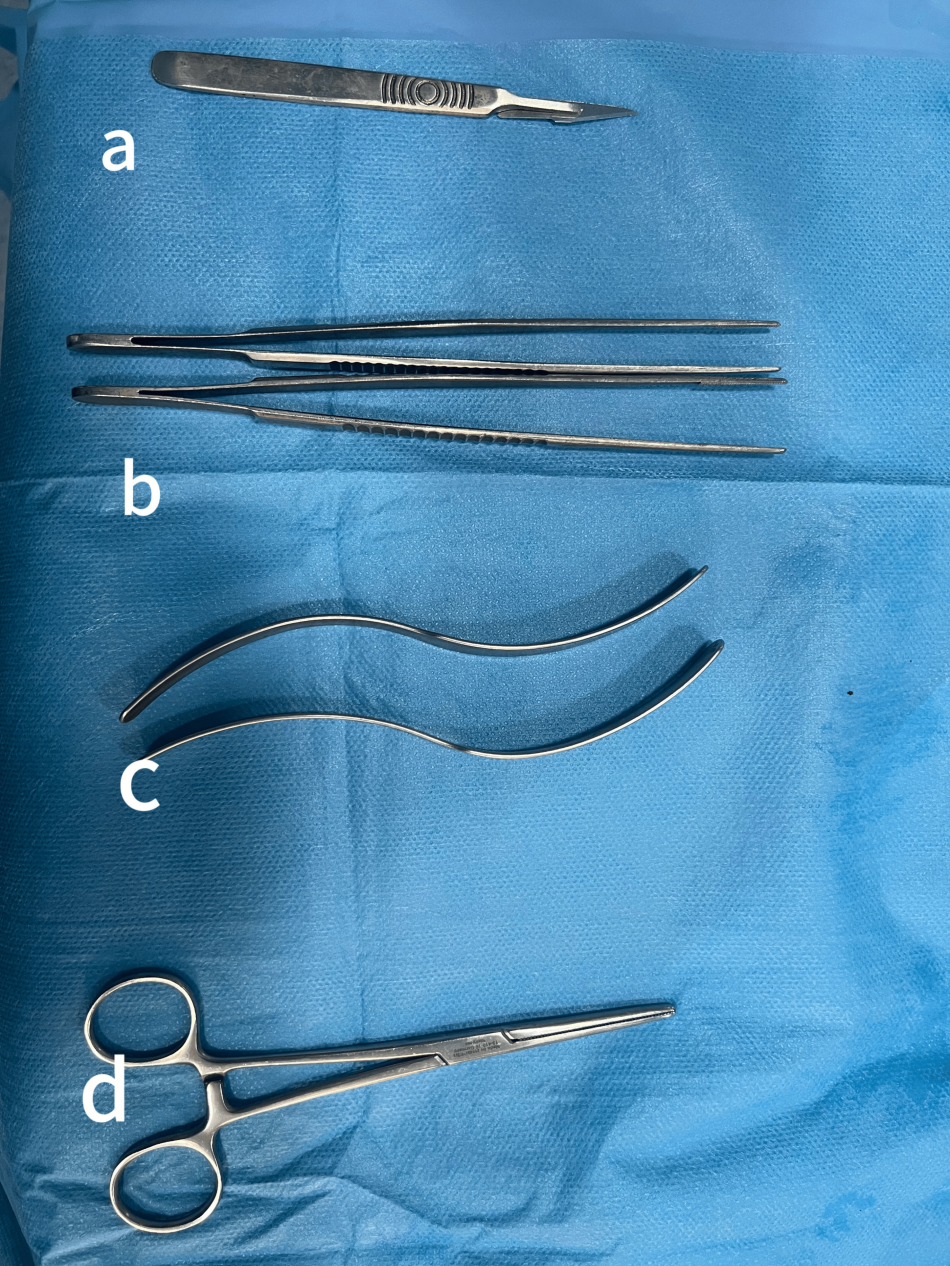
(a) Number 11 blade. (b) Debakey forceps. (c) S-shaped retractor. (d) Straight forceps with a round blunt tip.

This technique uses an S-shaped retractor with one large and one narrow extremity (Figure 1), a straight forceps with a round blunt tip, Debakey forceps, a number 11 blade, and a 10 mm blunt trocar (Figure 2).

**Figure 3:**
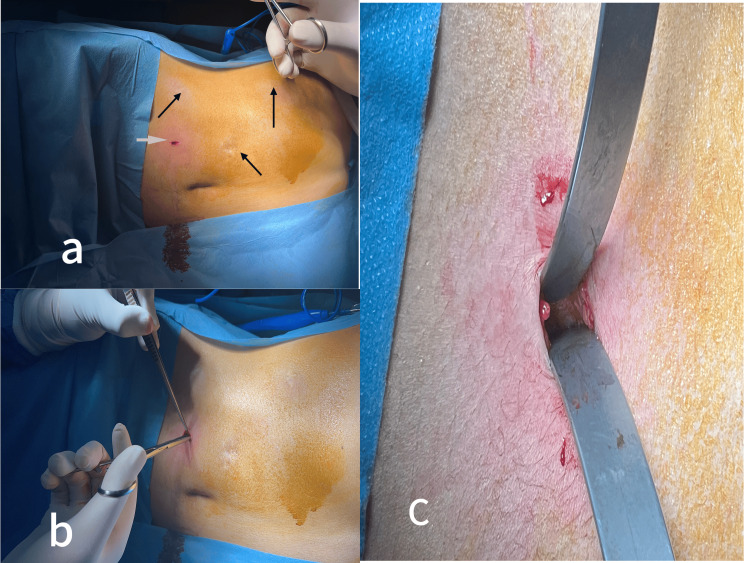
(a) Scars from previous laparoscopic surgery (black arrows) showing a 1.5 cm skin incision in a distant site from scars (white arrow). (b) Dissection of subcutaneous tissue using straight blunt forceps. (c) The aponeurosis is exposed using the large extremity of the S-shaped retractor.

After making a 1.5 cm skin incision, the aponeurosis was exposed using straight forceps and the large extremity of the S-shaped retractor (Figure 3).

**Figure 4:**
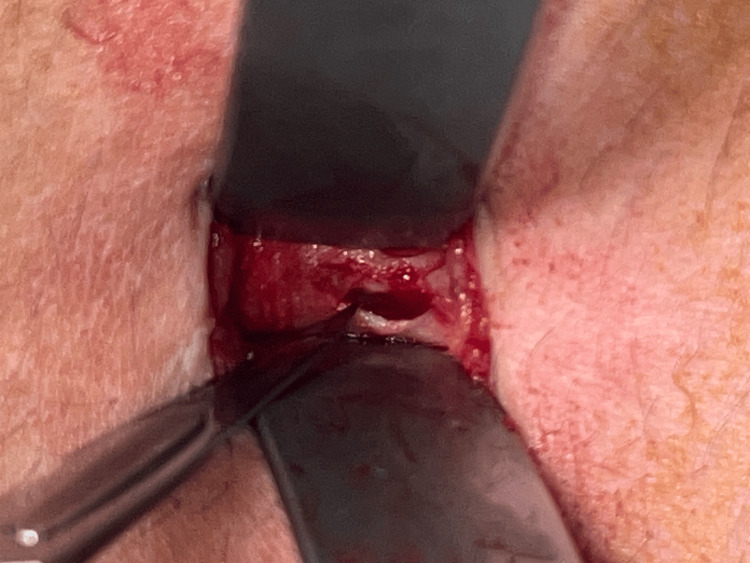
A small 10 mm incision is made in the aponeurosis using the tip of a No. 11 blade.

**Figure 5:**
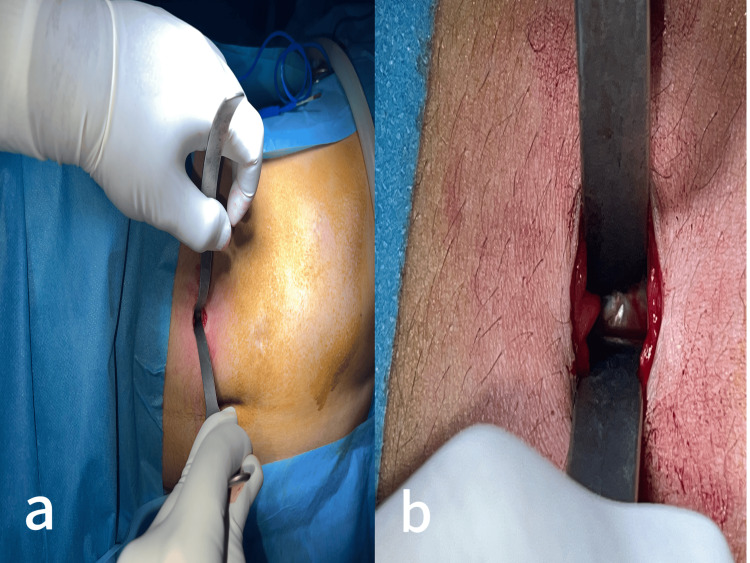
(a) The 10 mm segments of S-shaped retractors are inserted into the aponeurotic incision. (b) Exposition of the peritoneum.

A small 10 mm incision was made in the aponeurosis using the tip of a No. 11 blade (Figure 4). Then, the narrow segments of S-shaped retractors were inserted into the incision (Figure 5). The muscle fibers were gently spread apart until the peritoneum was reached (Figure 5). The latter was perforated using a round-tip forceps in an open-close, back-and-forth movement.

**Figure 6:**
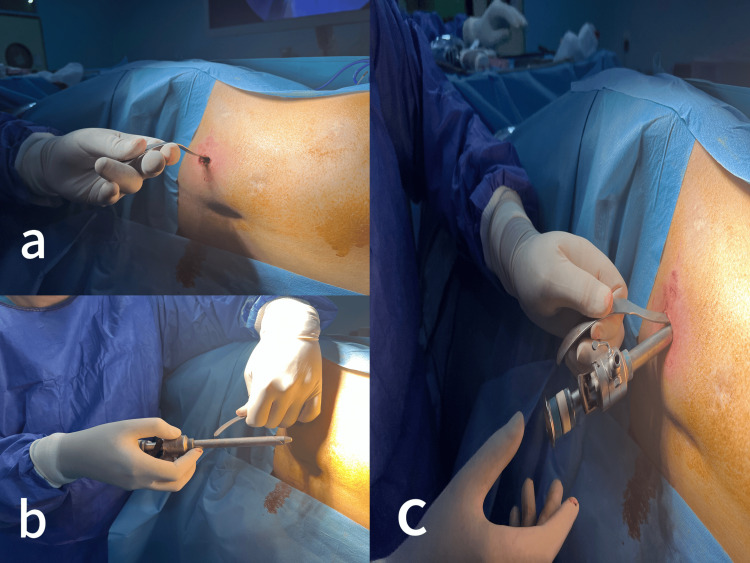
(a) The abdominal wall is lifted by the narrow part of the S retractor pushed into the peritoneal cavity. (b) The S retractor served as a guide for the blunt trocar. (c) The blunt trocar is introduced into the peritoneal cavity.

The narrow end of the S retractor was introduced into the abdomen through the small peritoneal opening. The abdominal wall was lifted by the narrow part of the S retractor (Figure 6), allowing the insertion of a 10 mm blunt trocar parallel to the narrow blade of the retractor (Figure 6).

After completing the procedure, this opening was left open and not closed.

We operated on 76 patients using this procedure and achieved a follow-up period ranging from four months to two years. The technique was applied effectively across three surgical specialties: urology, gynecology, and visceral surgery.

The technique was attempted in all regions of the abdomen: at the umbilicus, subumbilical area, along the pararectal line, and in the iliac fossa. In cases of scarred abdomens, the entry was made at a site distant from the scar. The results are summarized in Tables 1, 2.

**Table 1:** Classification and results of operated patients according to their BMI.

BMI range	Number of cases	% of total cases	Complications (Clavien-Dindo classification)	% of group with complications
20–30	77	83.7%	2 infections (grade 1), 1 omental tear (grade 1)	3.9%
>30	15	16.3%	1 infection (grade 1), subcutaneous hematoma (grade 1)	13.3%
Total	92	100%		
p-value				0.29

**Table 2:** Classification and results of operated patients according to their history of previous abdominal surgeries.

Scar abdomen (number of previous interventions)	Number	Complications (Clavien-Dindo classification)	Complication rate (%)	Percentage of total cases (%)	p-value
0	43	1 infection (grade 1)	2.33	56.58	
1	23	2 subcutaneous hematomas (grade 1)	8.70	30.26	
2	9	0	0.00	11.84	
3	1	0	0.00	1.32	
Overall chi-square p-value					0.56

Patients were classified according to their BMI and the number of previous abdominal surgeries. Postoperative complications were assessed according to the Clavien-Dindo classification, which stratifies surgical complications from grade I (minor deviation from the normal postoperative course) to grade V (death of the patient). Statistical differences between subgroups were assessed using a chi-square test of independence.

The results indicate that entry was always possible, and complications were rare, predominantly classified as Clavien-Dindo grade I. No cases of symptomatic clinical hernia at the trocar site were recorded.

## Discussion

Among the three primary laparoscopic entry techniques (Veress needle, direct trocar insertion, and the open Hasson technique), the open approach remains the most widely adopted, particularly in patients with prior abdominal surgeries or in complex cases where safety is paramount [2].

The Hasson technique has been extensively studied and consistently shown to be safe and reproducible across a range of surgical contexts [3]. Two key requirements underpin the success of this technique: the fascial opening must be sufficiently wide to allow smooth trocar insertion; simultaneously, the opening must be narrow enough to maintain pneumoperitoneum and provide stable fixation for the trocar.

This balance ensures a smooth continuation of the laparoscopic procedure without gas leakage or unintentional trocar dislodgement [4]. However, despite its advantages, the Hasson technique presents certain limitations and challenges that merit discussion.

A common criticism of the open technique is the need for fascial closure at the end of the procedure to prevent trocar site hernia. This step adds time to the operation, may increase the risk of wound complications, including infection, bleeding, or inadvertent injury to intra-abdominal structures [5], and often necessitates the use of specialized closure devices in some settings, which can raise procedural costs [6].

Opening the aponeurosis typically involves grasping it with two Kocher clamps, followed by sharp dissection. To access the peritoneum, a wide aponeurotic incision is required, which inherently increases the risk of gas leakage and subcutaneous emphysema [4].

Some surgeons attempt to mitigate this by placing anchoring sutures in the fascia before trocar insertion. While this approach can reduce leakage, it introduces additional steps and potential technical difficulty, especially in obese patients, where thick subcutaneous fat hampers suture placement and visibility [7].

Some authors propose the use of blunt-tip trocars with an inflatable balloon to ensure airtight fixation, which adds additional cost to the procedure [7].

In our surgical practice, we have observed that obese patients present significant challenges for the open technique, particularly at non-umbilical entry sites. Although not widely emphasized in the literature, the deeper abdominal wall and thick omental and preperitoneal fat layers in such patients obscure clear visualization of the peritoneum.

This difficulty necessitates the use of long, straight retractors to adequately expose the dissection site. Poor visualization increases the risk of entry complications and prolongs the access phase of the procedure [8].

To address these limitations, we have modified our approach to optimize efficiency and reduce complications. By limiting the size of both fascial and peritoneal incisions, we reduce the risk of gas leakage while eliminating the need for fascial sutures in most cases. This balance preserves pneumoperitoneum and secures trocar placement without additional instrumentation.

Rather than wide dissection or traction with multiple instruments, we use a single clamp to carefully dissect the peritoneum. This minimizes trauma and is especially advantageous in obese patients, where space and visibility are limited.

Omitting fascial closure at the end of the procedure simplifies the technique and effectively saves time. This decision was carefully made based on the small hole of aponeurosis and peritoneum, facilitated by the use of the 10 mm extremity of our S retractor. No clinical symptomatic hernia at the trocar site was recorded during the period of follow-up.

These refinements have resulted in shorter entry times, lower complication rates, and more streamlined procedures in our experience.

Challenging cases of obesity and scary abdomen were easily managed by our technique. Since our adoption of this technique, the entrance has been possible in all cases without significant complications.

Comparative studies with classical surgical techniques and prolonged follow-up are essential for a more comprehensive evaluation of outcomes. Imaging modalities may be necessary during follow-up to identify subclinical or occult hernias.

## Conclusions

The open Hasson technique remains a cornerstone of laparoscopic entry, particularly when safety is paramount. However, awareness of its limitations, especially in obese patients, and the adoption of refined, simplified approaches as our technique, may enhance procedural efficiency and safety. Surgeons should remain adaptable, employing techniques best suited to individual patient anatomy and operative context.

## References

[1] Shoman H, Sandler S, Peters A (2020). Safety and efficiency of gasless laparoscopy: a systematic review protocol. Syst Rev.

[2] Ahmad G, Baker J, Finnerty J, Phillips K, Watson A (2019). Laparoscopic entry techniques. Cochrane Database Syst Rev.

[3] Hasson HM (1971). A modified instrument and method for laparoscopy. Am J Obstet Gynecol.

[4] Thakore D, Ray MS, Modi N, Panchal B, Raval A, Patel V (2023). Comparative study of outcomes in patients where pneumoperitoneum is created by Veress needle versus open method in laparoscopic surgeries. Int Surg J.

[5] Güven E, Dura MC, Aktürk H, Güraslan H (2023). Safety of laparoscopic entry points in patients with a history of abdominal surgery: a research article. Cureus.

[6] Jeon Y, Song S, Han KW, Lee DH, Baek JH (2020). Evaluation of a novel trocar-site closure device in laparoscopic surgery. JSLS.

[7] Polat M, Incebiyik A, Tammo O (2023). Abdominal access in laparoscopic surgery of obese patients: a novel abdominal access technique. Ann Saudi Med.

[8] Miti C, Busuulwa P, Scott R, Bloomfield-Gadelha H (2023). Primary entry trocar design and entry-related complications at laparoscopy in obese patients: meta-analysis. BJS Open.

